# Computer Vision-Based Microcalcification Detection in Digital Mammograms Using Fully Connected Depthwise Separable Convolutional Neural Network

**DOI:** 10.3390/s21144854

**Published:** 2021-07-16

**Authors:** Khalil ur Rehman, Jianqiang Li, Yan Pei, Anaa Yasin, Saqib Ali, Tariq Mahmood

**Affiliations:** 1 The School of Software Engineering, Beijing University of Technology, Beijing 100024, China; rehmankhalilur@emails.bjut.edu.cn (K.u.R.); lijianqiang@bjut.edu.cn (J.L.); yasinanaa@emails.bjut.edu.cn (A.Y.); saqibsaleem788@hotmail.com (S.A.); khalil_ur@hotmail.com (T.M.); 2 Beijing Engineering Research Center for IoT Software and Systems, Beijing 100124, China; 3 Computer Science Division, University of Aizu, Aizuwakamatsu, Fukushima 965-8580, Japan; 4 Division of Science and Technology, University of Education, Lahore 54000, Pakistan

**Keywords:** microcalcification detection, image processing, fully connected depthwise convolutional neural network, breast cancer

## Abstract

Microcalcification clusters in mammograms are one of the major signs of breast cancer. However, the detection of microcalcifications from mammograms is a challenging task for radiologists due to their tiny size and scattered location inside a denser breast composition. Automatic CAD systems need to predict breast cancer at the early stages to support clinical work. The intercluster gap, noise between individual MCs, and individual object’s location can affect the classification performance, which may reduce the true-positive rate. In this study, we propose a computer-vision-based FC-DSCNN CAD system for the detection of microcalcification clusters from mammograms and classification into malignant and benign classes. The computer vision method automatically controls the noise and background color contrast and directly detects the MC object from mammograms, which increases the classification performance of the neural network. The breast cancer classification framework has four steps: image preprocessing and augmentation, RGB to grayscale channel transformation, microcalcification region segmentation, and MC ROI classification using FC-DSCNN to predict malignant and benign cases. The proposed method was evaluated on 3568 DDSM and 2885 PINUM mammogram images with automatic feature extraction, obtaining a score of 0.97 with a 2.35 and 0.99 true-positive ratio with 2.45 false positives per image, respectively. Experimental results demonstrated that the performance of the proposed method remains higher than the traditional and previous approaches.

## 1. Introduction

Breast cancer is the leading cancer resulting in death among women around the globe. The mortality rates are very high in Asia and Africa compared to Europe according to the World Health Organization International Agency for Research on Cancer (WHO-IRAC) [1]. The automatic detection of suspected lesions from mammograms at the early stages could help radiologists in the prediction of breast cancer in less time to avoid unnecessary biopsies in order to reduce mortality rates [2]. Mammography is a standard screening method for the detection of breast cancer, but it is not a golden standard for breast cancer diagnostics. Moreover, mammography is an effective tool for the detection of breast cancer in women at the early stages. Microcalcification develops in the breast and is one of the main signs of breast cancer visible on mammograms at the early stages. Microcalcifications are tiny tissues having a diameter from 0.1 mm to 0.7 mm that comprise features such as shape, morphology, and location, which can be extracted in the phase of preprocessing to differentiate malignant from benign lesions [3]. Therefore, the manual recognition of microcalcification clusters is very difficult for radiologists during the examination of mammograms due to their low contrast, small size, shape, and scattered location inside the denser masses. Microcalcifications are more likely to be benign if the diameter is 0.1 mm or under 0.5 mm, while a heterogeneous diameter smaller than 0.5 mm is malignant [4].

Radiologists manually examine the presence of microcalcification clusters in mammograms to predict breast cancer at the initial stages, increasing the false-positive rate of the actual positive malignant cases. In the literature, to overcome these human errors and enable the automatic detection of microcalcification clusters, there has been great effort to develop a computer-aided diagnostic system. These methods successfully achieve a higher sensitivity rate and decrease the false-positive rate. Several factors are involved in the occurrence of FPs, such as noise, imaging artifacts, the tiny size of MCs, and the linear background structure. Therefore, mammogram screening using a computerized model can identify suspicious regions in the mammogram. Therefore, it is challenging to maintain a higher true-positive rate and a lower false-positive rate. Reference [5] developed a CAD based on deep learning modalities for the screening of mammograms to predict breast cancer. Traditionally, breast cancer prediction from a digital mammogram has to two steps. In the first step, the microcalcification clusters are detected by extracting the ROIs (regions of interest) to perform segmentation from the mammogram images. In the second step, the clustered ROIs are categorized into malignant and benign classes for the prediction of breast cancer.

Several methods were employed to develop CAD systems in the previous studies, such as the enhancement, edge detection, wavelet transform, microcalcification detection, and ROI segmentation of images and then classifying them into malignant and benign. There are many limitations to these methods, for example: Hadijidj et al. [6] proposed a watershed morphology operation to extract the segmented regions from mammograms for the detection of microcalcifications. In addition, the watershed morphology technique was employed for edge detection and region growing, while noise removal was still a problem. Juan W. et al. [7] proposed a direct global detection approach for clustered MCs in a mammogram and then input these clusters into a deep neural classifier for classification. With this approach, the classifier was limitedin the identification of individual local MC regions. Wang J. et al. [8] developed a different approach to detecting the presence of clustered MCs in mammograms instead of first detecting the MCs individually. The clustered MCs were directly input into the CNN classifier for classification. The intercluster gap, noise between individual MCs, and individual object location can affect the classification performance, which may reduce the true-positive rate. A variety of conventional methods were employed to extract handcrafted features from mammograms such as morphology, shape, and texture, and then, machine learning algorithms were applied for the prediction of malignant and benign microcalcifications from the clustered MCs. Manual feature extraction and classification with machine learning require much time, which decreases the performance of the model. Therefore, the handcrafted feature extraction methods ignore the essential attributes of images for the extraction of microcalcification ROIs. Moreover, the traditional deep learning neural network has very low classification performance, which affects the sensitivity of the cancer detection rate [8]. Among all the above limitations, the detection of microcalcification clusters from digital mammogram images using traditional approaches can affect breast cancer prediction and increase false-positive cases.

In this study, we investigate the research gap by using the computer vision method for the detection of MCs from digital mammograms. With this approach, there is no longer a limitation in microcalcification detection. The computer vision method automatically controls the noise and background color contrast and directly detects MC objects from mammograms, which increases the classification performance of the neural network. Computer vision is a powerful technology for object detection and feature extraction. Computer vision algorithms can improve the intelligence, functionality, interaction, and efficiency of objection detection. In addition, computer vision technology can reduce the noise and control the color contrast and brightness of images during preprocessing and feature extraction. Y. Tang et al. [9] conducted a critical review of the recognition capability and research applications of computer vision technology in fruit picking. Moreover, in the second phase, we address the limitations of traditional deep neural networks in classifying these MC clusters into malignant and benign classes. The depthwise-separable convolutional neural network uses the spatial dimension for the classification, which increases the learning capability of the network that performs the classification, achieving an accurate prediction in fewer epochs.

We aim to develop an automated CAD system based on computer vision and deep learning to detect and classify MC clusters to discriminate the malignant and benign classes and increase the sensitivity. This study proposes a novel diagnostic method based on computer vision and a fully connected, depthwise-separable convolutional neural network (FC-DSCNN) to detect microcalcification clusters and classify these clusters to predict breast cancer at the early stages. Moreover, our proposed fully automated computer-vision-based diagnostic system correctly detects microcalcification clusters in comparison to previous studies, which increases the breast cancer prediction rate. The proposed method can achieve a better sensitivity rate compared to the traditional DCNN and previous studies. We further evaluated the performance of our proposed method with other metrics such as the F1-score, recall, and area under the receiver operating characteristic curve (AUC). The principal outcomes of this study are as follows:

- We propose a fully automated deep-learning-based computer-aided diagnosis system for the prediction of breast cancer from a digital mammogram. In the first step, the mammogram is segmented into subregions using a computer-vision-based method to extract MC clusters to locate the suspicious regions;
- In the second phase, the fully connected depthwise-separable CNN is used to classify these segmented MC clusters into malignant and benign classes. We also use the traditional deep 2D CNN for comparing our proposed method on the same procedure;
- In the diagnosis phase, the fully automated computer-vision-based FC-DSCNN predicts breast cancer from digital mammograms;
- Our proposed method can achieve a better sensitivity compared to previous studies. Furthermore, the evaluation metrics such as the recall, F1-score, and AUC curve are calculated to verify the model’s diagnostic ability;
- Moreover, in this study, we evaluated for the first time the local PINUM dataset and the public DDSM dataset for the prediction of breast cancer with our proposed approach and achieved a higher true-positive rate;
- The evaluation of the local dataset could help doctors and radiologists diagnose cases of breast cancer in women at the initial stages in real time.

The rest of the paper is structured as follows. Section 2 describes the existing literature. Section 3 contains the materials and methods. Section 4 discusses the experimental results. In Section 5, we discuss our findings, followed by Section 6, where we give the conclusion and future work of this study.

## 2. Literature Review

Several computer-aided diagnosis systems have been proposed by researchers for the prediction of breast cancer from mammogram images at the early stages. Many studies employing machine learning and deep learning algorithms for the classification of breast cancer are herein briefly presented. The main purpose of this study is to carry out a summary of the existing work related to breast cancer detection based on microcalcification clusters and classification using a deep convolutional neural network. In recent years, deep learning networks have been applied in the classification of digital medical images for object detection and disease prediction. Moreover, we also summarize those studies that used handcrafted feature extraction techniques for breast cancer detection using deep learning and machine learning.

In this study, we consider the deep neural network as a classification model; therefore, we also include those studies that employed convolutional neural networks (CNNs) for automatic feature extraction from images and classification for the prediction of breast cancer. Juan W. et al. [7] applied a global detection approach for microcalcification detection from digital mammograms, and a deep learning neural network was employed as a classifier model. A context-sensitive deep learning model was employed for the detection of microcalcification from mammograms [8]. G. Valvano et al. [10] developed a novel approach based on a deep convolutional neural network for the detection of microcalcification clusters from digital mammograms. Microcalcification clusters in mammography indicate the early signs of cancer. H. Cai et al. [11] employed a deep convolutional neural network for breast microcalcification diagnosis. A computer-aided microcalcification detection model was developed using the firefly algorithm and extreme learning by S. R et al. [12]. Mabroul et al. [13] developed a breast cancer diagnostic system for microcalcification detection from mammograms based on shape features.

Furthermore, we briefly summarize those studies with different modalities for breast cancer prediction based on microcalcification clusters. Jayesh G. M. and Perumal S. S. [14] proposed a hybrid extreme learning machine algorithm for the automatic detection of microcalcification from digital mammogram images and employed machine learning algorithms for the classification of MCs. A context-sensitive classification model was developed for the detection of clustered microcalcification from digital mammograms to improve the accuracy by [15]. X. Zhang et al. [16] presented a hybrid model using morphology and the wavelet transform technique for the detection of microcalcification clusters from digital mammograms. A fuzzy-c-means algorithm was employed for the detection of microcalcification clusters from digital mammograms by L. Vivona et al. [17] and to improve the sensitivity rate. J. Wang et al. [18] employed deep learning for the discrimination of breast cancer based on microcalcifications. The independent component analysis technique was used for the detection of microcalcification clusters to predict breast cancer and improve the sensitivity rate by [19]. A fully automated computer-aided system was presented using the Hough transform for the detection of microcalcification clusters by T. M. A. Basile et al. [20] from digital mammograms, and a clustering algorithm was employed for classification.

Unfortunately, handcrafted feature extraction and then classification with a convolutional neural network approach are computationally heavy, which reduces the sensitivity of cancer detection. Therefore, we also critically analyzed those studies that pertain to deep learning techniques for breast cancer prediction using handcrafted and mass features. Deep CNN was applied using the deep contextual information imaging modality to predict breast cancer from mammograms [21]. An improved deep convolutional neural network was applied for the classification of digital mammograms and achieved a significant improvement in the cancer detection rate [22]. R. Suresh et al. [23] developed a CAD for the detection of abnormal and normal patterns from mammograms using a deep neural network to increase the accuracy of breast classification. The Faster-RCNN was developed [24] for regional classification in order to increase the sensitivity of mass detection, and therefore the rate of detection, from mammograms for the prediction of breast cancer. Reference [25] developed an approach based on a deep learning algorithm for the classification of breast masses and achieved an accuracy of 96.47 by evaluating the approach on public datasets. An extreme learning approach was employed to map the feature fusion to obtain the CNN features for breast cancer detection and classification by Z. Wang et al. [26].

A hybrid model was proposed using deep learning for the classification of mammogram screening exams read by expert radiologists to evaluate the model accuracy and cancer detection rate [27]. A multiview-feature-fusion-based mammogram classification model based on a deep learning neural network was presented [28] for the detection of mass and calcification from mammograms. Mammogram segmentation is a challenging task to determine suspicious regions. H. Soleimani et al. [29] employed the segmentation of pectoral muscle to locate cancerous regions in mammograms and used a deep learning algorithm for the classification. A deep learning data-driven-based approach was proposed [30] for automatic identification of cancerous regions from mammograms, which improved the performance of the classification. D. song et al. [31] applied a deep neural network for breast cancer prognosis prediction from multidimensional data and achieved a specificity of 99%. Multilayer perceptron (MLP) is a feed-forward neural network class that has three layers: the input layer, the hidden layer, followed by the output layer, which was compared [32] with other algorithms to improve the model accuracy for the prediction of breast cancer from fine-needle aspiration. Xin Shu et al. [33] proposed a region-based pooling structure deep neural network for mammogram image classification. In [34], a novel deep neural network approach was presented with a mapping technique for the neuron structure for the classification of cancerous and noncancerous mammograms. To overcome the distance between the neurons, self-organizing map was used [35] to reduce the training time, and the classification task was performed with an artificial neural network. Kui Liu et al. [36] presented a fully connected layerconvolutional neural network to enhance the performance of the classification on the Wisconsin Breast Cancer Diagnostic Database (WDBC) dataset and achieved higher accuracy.

Finally, we briefly summarize some studies that presented various breast cancer prediction computer-aided diagnosis systems. Gem Tekin et al. [37] proposed a novel image stream mining technique for the classification of live streaming images and evaluated the result using the AdaBoost algorithm, achieving significant improvement in the prediction rate. Zhang et al. [38] developed a CAD based on a Gaussian filter to enhance the image texture and remove the noise for the prediction of breast cancer. In [39], the GLCM feature was extracted using fuzzy-c-means from mammograms. and the classification task was applied using a support vector machine. R. Shen et al. [40] presented a novel active learning self-paced algorithm, minimizing the annotation task for the detection of breast cancer from mammograms. In [41], a stable extremal region detector was applied for the classification of mammograms into malignant and benign. F. Mohanty et al. [42] employed a hybrid approach using the C4.5 algorithm to detect the suspicious regions from mammograms for the classification of cancerous and noncancerous cases. A case-based reasoning technique for the visualization of a structured dataset [43] was applied for the classification of breast cancer images into a binary class. The K-nearest neighbor algorithm was used for the classification of wavelet features into normal and abnormal images [44]. To improve the performance of mammogram classification, Wang H. et al. [45] employed a hybrid deep convolutional neural network and attained remarkable improvements in their evaluation results. L. Sun et al. [46] employed a mass detection from mammography technique based on a template matching deep convolutional neural network. M. I. Daoud et al. [47] presented a breast tumor classification method using a hybrid deep convolutional neural network for breast ultrasound images. Hiren K M. et al. [48] proposed a technique for breast cancer classification using a spatial feature integrated neural network. It was shown that the regional and diffused distributions of the calcification are typically benign, while the clustered and segmental distributions are malignant [49]. A. Bala et al. [50] presented an automatic computer-aided diagnostic system for the detection of suspicious microcalcification lesions from breast mammograms.

## 3. Materials and Method

**Figure 1:**
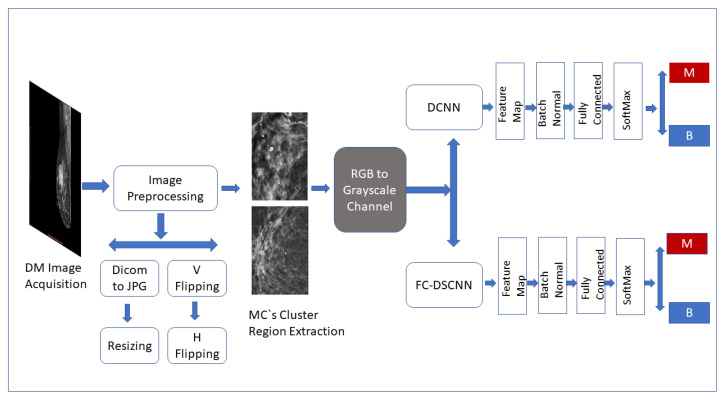
Framework of the proposed CAD system. The breast cancer classification framework has four steps: image preprocessing and augmentation, microcalcification ROI segmentation, RGB to grayscale channel conversion, and microcalcification cluster classification to predict malignant and benign cases.

The CAD framework of the proposed method FC-DSCNN along with the DCNN for microcalcification classification are presented in Figure 1. The breast cancer classification framework had four steps: image preprocessing and augmentation, microcalcification ROI segmentation, RGB to grayscale channel conversion, and microcalcification cluster classification to predict malignant and benign cases. The details of the proposed methodology are given in the subsequent sections.

### 3.1. Dataset

**Figure 2:**
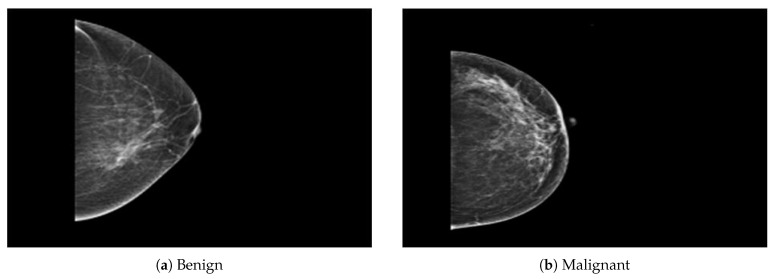
An example of breast mammogram images from the PINUM dataset. (**a**) The benign image. (**b**) The malignant image. The images were labeled by the expert radiologist in the dataset.

**Figure 3:**
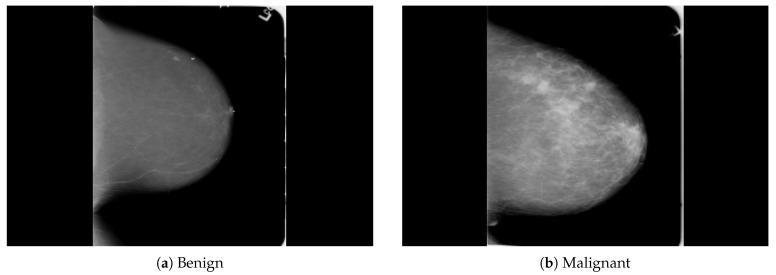
An example of breast mammogram images from the DDSM dataset. (**a**) The benign image. (**b**) The malignant image. The images were labeled with verified pathology information.

The dataset was collected from a local (PINUM) hospital [51] in Pakistan, with the approval of the Diagnostic Imaging Nuclear Medicine and Radiology Research and Development Committee. A total of 289 patients were manually labeled by the expert radiologist using the initial mammogram reports generated by the mammography machine and included ages between 32 and 73 with a mean age of 48.5 years. The radiologist team consisted of two members, one being a senior radiologist and physicist holding a PhD degree in nuclear medicine with 10 years of experience and the second being a junior radiologist with a Master’s degree in radiology. The dataset was first evaluated with the proposed method to increase the sensitivity of breast cancer prediction using a fully automated CAD system. The dataset included 577 original images consisting of 425 benign and 152 malignant images with both MLO (mediolateral-oblique) and CC (craniocaudal) views at a resolution of 4096 × 2047, as shown in Figure 2. Each CC and MLO view image had a focal length of 35 mm with a 96 dpi resolution along the horizontal and vertical axis. The mammography exam of the PINUM dataset was acquired with Hologic 2D, 3D mammography. Data augmentation techniques were employed on the PINUM dataset to increase the size to regularize and train the deep convolutional neural network. Each mammogram image was rotated at four angles, obtaining five images, including one original and four rotated images. Moreover, we included another public dataset, the Digital Database for Screening Mammography (DDSM) [52], for the evaluation of our model, which contains 3568 mammogram images (1740 benign, 1828 malignant) from 870 benign and 914 malignant cases, as shown in Figure 3. We split the dataset into 60:20:20, where we used 60% for the training of our model, 20% for cross-validation, and the rest for testing. We used 5-fold cross-validation to test our dataset. A detailed description of the dataset is given in Table 1.

### 3.2. Image Preprocessing and Augmentation

The PINUM database was used first to evaluate the proposed method’s efficiency at diagnosing breast cancer and achieving a significant improvement in the sensitivity rate. The acquisition of the original mammogram was in Digital Imaging and Communications in Medicine (DICOM) format, so that automatic preprocessing was applied to convert the images into PNG format to avoid the loss of the pixel values. We used the computer-vision-based method to preprocess the DICOM images into PNG format and extract all the patient information into a CSV file.

The converted PNG breast mammogram images had a high resolution of 4096 × 2047 (width and height). To resize the images, we employed an automatic resizing method to maintain the original pixel values. The OpenCV resize method was applied to preserve the aspect ratio by calculating the width and height, respectively. Moreover, the INTER_LINEAR interpolation technique was used to obtain the pixel values of the neighboring pixels. As a result, the newly resized images were acquired at a resolution of 320 × 240 (width and height). The complete steps of the image preprocessing Algorithm 1 are reported below.

**Algorithm 1** Image preprocessing algorithm.**Ensure:** Acquisition = Original DICOM mammogram images;**Ensure:** Output = Mammogram image; 1. Acquire DICOM images; 2. Read image description; 3. Write image description into the pixel array; 4. Write patient description into the CSV file; 5. Replace the pixel array of DICOM with the PNG format; 6. Write the new format, and return the PNG image; 7. Read the image using cv2-imread, and store the pixels in an array; 8. Scale the image to the original aspect ratio; 9. Input the original dimension of the image; 10. Input the new width and height with the (320 × 240) dsize method; 11. Set the new dimension of the original image, and store it in the array; 12. Output the new resize of the image and save the image.

**Figure 4:**
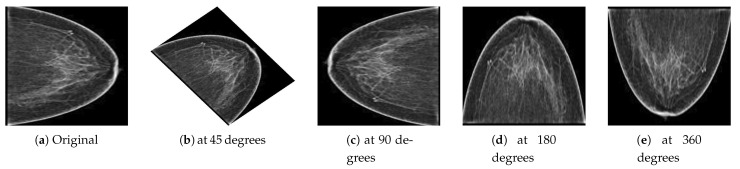
(**a**) Original image and the resultant images from the PINUM dataset after the augmentation steps were applied to achieve the rotated images (**b**) at 45 degrees, (**c**) at 90 degrees, (**d**) at 180 degrees, and (**e**) at 360 degrees.

Deep learning is a data-driven approach; therefore, many images were required to train the network to classify malignant and benign images. However, to handle the generalization and overfitting and improve the robustness of the deep learning model, we artificially inflated the PINUM database four times from the original images to increase the dataset size. The data augmentation techniques of vertical flipping, horizontal flipping, rotating, and image cropping were employed to increase the dataset. The mammogram images were rotated at 45, 90, 180, and 360 degrees to return a new object of the rotated images within a described resolution to increase the dataset size to 2885. The computer vision method was employed to keep the original dimensions of the images and fit them into the frame with the rotated angle, as shown in Figure 4. Guan et al. [53] applied image augmentation methods such as vertical flipping, horizontal flipping, shifting, scaling rotation, width shifting, and shearing to help generalize the deep convolutional neural network for the classification of synthetic mammogram images. The acquisition of DDSM mammograms was in DICOM format at a very high resolution. We employed the same method on the DDSM dataset for the image preprocessing. The size of the DDSM dataset is 3568 images; therefore, data augmentation was not employed on the DDSM dataset.

### 3.3. Microcalcification ROI Segmentation

**Figure 5:**
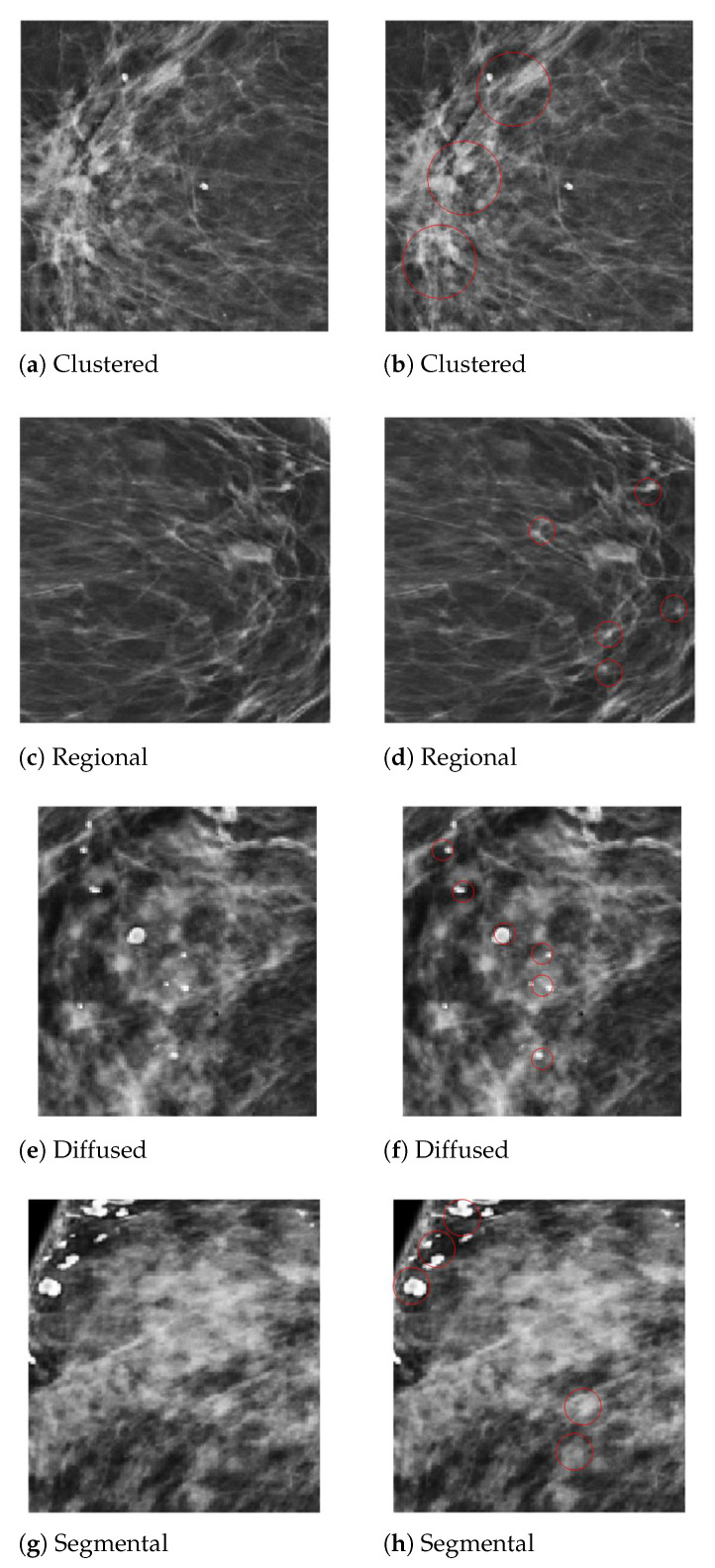
An example of the microcalcification ROI segmentation of the PINUM dataset using computer vision. (**a**) Clustered MC ROI without a mark. (**b**) Marked clustered malignant MC ROI. (**c**) Regional MC ROI without a mark. (**d**) Marked regional benign MC ROI. (**e**) Diffused MC ROI without a mark. (**f**) Marked diffused benign MC ROI. (**g**) Segmental MC ROI without a mark. (**h**) Marked segmental malignant MC ROI.

In the diagnosis of breast cancer, the analysis of the calcifications’ shapes and their distribution in the breast composition is the most important factor in differentiating a malignant from a benign mammogram. The detection of MC clusters from a mammogram is a challenging task due to their tiny size. We employed two steps for MC cluster detection, region of interest (ROI) segmentation with imagewise labeling. In the first step, the areas considered to be the ROIs were those whose diameter was between 0.1 mm to 0.5 mm, having a heterogeneous shape and labeled by expert radiologists, to locate the clustered MCs in digital mammograms. In the second step, we used OpenCV to extract the ROI images’ numerical pixel values and then create an image pixel array. We used the image translation for the shifting of image pixels around the (x,y) direction. The translation matrix *M* was used to shift the pixel in the (x,y) direction as ($B_{x}$, $M_{y}$), where $B_{x}$ are benign MCs pixel and $M_{y}$ are malignant pixels, as shown in Equation (1). In the second step, the pixel matrix was labeled with benign and malignant MC clusters by creating an input vector for a fully connected depthwise-separable convolutional neural network. Figure 5 presents the segmented MCs’ ROIs from the PINUM dataset mammograms with individual MCs marked by a circle. Moreover, we employed the same procedure on the DDSM dataset for MCs’ ROI extraction.

(1) $$M=\left[\begin{array}{cc} 1 & 0\\ 0 & 1 \end{array}\right]\left[\begin{array}{c} B_{x}\\ M_{y} \end{array}\right]$$

### 3.4. RGB to Grayscale Channel Conversion

The mammogram is a grayscale image with one color channel, and it has only one dimension. We used OpenCV to read the grayscale images, which automatically transforms images into red, green, and blue channels. To keep each color channel’s grayscale value, we converted the RGB image into grayscale. The average method was employed to convert the RGB to the grayscale channel, as shown in Equations (2) and (3). WE initialized the model using the channel first to create the input shape and then reshaped the images into three dimensions, width, height, and depth, by updating the channel dimensions. We built our deep neural networks using the input reshape class in three dimensions: depth, width, and height. Image classification with one color channel was not a limitation of the deep neural network. However, the depthwise-separable CNN uses the spatial dimension of images, which increases the network’s learning capability, improving the prediction performance in fewer epochs.

(2) $$G=\frac{\left(r+g+b\right)}{3}$$

(3) $$NG=(\left(r*P_{r}\right)+\left(g*P_{g}\right)+\left(b*P_{b}\right))$$

where *G* is a grayscale image and converted to RGB color, *r* is red, *g* is green, and *b* is blue. NG is a new grayscale image for conversion. The three colors have different wavelengths and pixel value percentages. $P_{r}$ is the pixel value percentage for red, $P_{g}$ for green, and $P_{b}$ for blue.

### 3.5. Proposed Method (FC-DSCNN)

The DCNN is a basic architecture for computer vision and medical image processing, having a single channel convolutional layer that has a very low sensitivity rate for the detection of breast cancer from mammogram images. To analyze the mammographic structure, we propose a novel fully connected depthwise-separable deep convolutional neural network-based CAD system for the detection of microcalcification ROIs from mammogram images and classification into malignant and benign masses, achieving a better sensitivity compared to the DCNN and with the previous studies.

**Figure 6:**
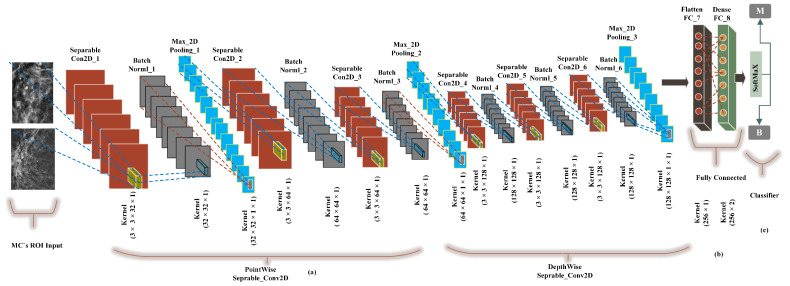
The implementation of the proposed FC-DSCNN. (**a**) The 3 × 3 Separable2D convolutional layer performs depthwise-separable convolution, which is followed by batch normalization, followed by the 2 × 2 max-pooling layer, (**b**) A fully connected flatten layer as the encoder and the dense layer. (**c**) The softmax classifier is used for the classification of MC ROIs into the benign and malignant classes.

We built a CancerNet FC-DSCNN model similar to VGGNet-16-19, and the implementation process of the proposed architecture is presented in Figure 6 with an eight-layer network consisting of six separable convolutional layers (separable_Con_2D) and two fully connected (FC) layers. The depthwise-separable convolutional layer deals with the spatial dimension, the number of color channels of the images, that is the width, height, and depth, and each channel has a particular interpretation of the image. The depthwise-separable convolution splits the kernel into two separate convolutions, depthwise and pointwise, performing six convolutions. The pointwise-separable convolution uses a 3 × 3 × 1 kernel that iterates through every single point and transforms images into a 3 × 3 × 1 × 1 feature map. Depthwise separable convolution employs three kernels of shape 3 × 3 × 1 and iterates one channel of the image, producing the scalar product of each pixel by creating a 3 × 3 × 1 × 1 feature map. We uses 6 (32, 64, 64, 128, 128, 128) convolutional layers for feature extraction.

Moreover, after each convolution, the batch normalization layers respond to the output of the first convolution and transform the feature map into the final shape with a max-pooling layer with a kernel size of 2 × 2. The output of each filter is a feature map. This is the first use of depthwise-separable convolution for mammogram classification in deep learning. We used a standard rectified linear unit (ReLU) as the activation function. Finally, two fully connected flatten and dense layers were used as the encoder, which had better performance, creating a 256-feature vector with two neurons, respectively. In the final output, the softmax classifier was used for the classification of MC clusters into malignant and benign classes, achieving a higher sensitivity rate as a result of the model evaluation. Wang et al. [54] pretrained AlexNet and Resnet50 for the prediction of breast cancer, which achieved a decent improvement in the Dice index.

### 3.6. FC-DSCNN Training

In our FC-DSCNN model, we split our dataset into 60% for the training set, 20% for testing, and 20% for validation in the 5-fold cross-validation. After the data augmentation, the PINUM dataset contained 2885 digital breast mammogram images with a size of 320 × 240 pixels, both CC and MLO views, from which 2125 were negative and 760 positive images. The PINUM private dataset was collected from a local hospital and used for the first time in breast cancer prediction. The DDSM dataset consists of 3568 images of 320 × 240 pixels, including 1740 benign and 1828 malignant images. Each image has a specific class label, class_0 for benign and class_1 for malignant. We built a depthwise-separable CNN and called it CancerNet, similar to VGGNet, which performs 3 × 3 convolutions and batch normalization before performing max-pooling. The network structure considered in the experiments is summarized in Table 2.

The three parameters, width, height, and depth, were the input for the shape of the image and contained each color channel of the image. The fully connected layers and softmax classifier predicted the microcalcification clusters as malignant and benign classes. We set 20 epochs to reduce the learning rate by a factor 0.1 after every 5 epochs, with the batch size being 32, and the loss function (sparse_categorical_crossentropy) was used to deal with the training data imbalance. The data augmentation object, trainAug, was initialized to apply random rotations, flipping on large datasets, and the model was successfully trained to evaluate the testing data and then validated with the corresponding predicted labels.

### 3.7. Deep CNN

**Figure 7:**
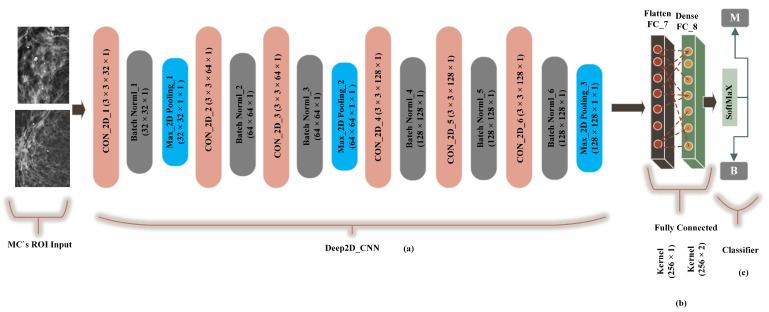
The implementation of the DCNN. (**a**) The 3 × 3 Con_2D convolutional layer, which is followed by batch normalization, followed by the max-pooling layer. (**b**) A fully connected flatten layer as the encoder and the dense layer. (**c**) The softmax classifier is used for the classification of malignant and benign MCs.

A traditional deep convolutional neural network-based computer-aided system presented by G. V Ionescu et al [55] was modified to 2-dimensional convolutions and compared with the proposed method (FC-DSCNN). The preprocessing step was the same as the FC-DSCNN. We built a Cancer2DNet DCNN model similar to VGGNet-16-19, and the architecture is presented in Figure 7 with an 8-layer network consisting of 6 2D convolutional layers (Con_2D) and 2 fully connected (FC) layers. We used 63 × 3 × 1 2D convolutions for feature extraction from the MC ROIs. The 2-dimensional convolutions were transformed into images in batch normalization followed by a 2 × 2 max-pooling layer. The fully connected (flatten, dense) layer created a 256 feature vector with 2 neurons, respectively. We used a standard rectified linear unit (ReLU) as the activation function. Finally, the softmax classifier predicted the microcalcification clusters as the benign and malignant classes.

### 3.8. Training the DCNN

A total of 2885 PINUM dataset images and 3568 DDSM mammogram images were employed for training, testing, and validation with a ratio of 60:20:20. E. K Kim et al. [56] applied a 4-layer deep convolutional neural network to train on the digital mammograms and evaluated the network’s ability to detect cancerous images. The PINUM local dataset was evaluated for the first time using the DCNN for the detection of microcalcification clusters as benign and malignant classes. Similar parameters, the width, height, and depth, as the FC-DSCNN were employed to take the input shape of the images with separate color channels, and the model was successfully trained and evaluated with the predicted labels. The class labels and the resolution of the images for both datasets were the same as for our proposed method. The detailed structure of the DCNN is described in Table 3. We performed 20 epochs with the same batch size as in our proposed method.

### 3.9. Deep Neural Network Regularization by the Training Process

Deep learning is a data-driven approach, and the generalization ability of the model can determine the effectiveness of the neural network. We performed two kinds of data augmentation to handle the regularization and overfitting issues. In the first step, we applied data augmentation on our dataset to handle the regularization of the deep neural network. However, the TensorFlow/Keras library provides a .fit_genrator method to accomplish data augmentation when training the model. In the second phase, to improve the regularization of our deep neural network, the data augmentation method (.fit_genrator) was employed on our network. The method .fit_genrator automatically applies augmentation steps, such as random rotation, shifts, shears, and flips, when the network is trained.

### 3.10. Performance Evaluation

The proposed method was evaluated on the local PINUM dataset and on the DDSM dataset to assess its performance using the following evaluation metrics: sensitivity, specificity, F1-sore, precision, recall, FPi, area under the curve (AUC), and accuracy. The proposed method detected the microcalcification clusters and significantly improved the sensitivity with a better false-positive index. The sensitivity showed that it correctly performed the true positive, true negative, and precision classification, indicating the actual predicted positive cases. The F1-score is the measurement of the tested accuracy, and it can be calculated to compute the precision and recall. Precision is the proportion of the positive predicted values, also known as the positive predictive value (PPV).

The average rate of false-positive cases actually predicted from the total cases is the rate of false-positives per image (FPi). The AUC curve was calculated, which indicates the ratio between the true-positive rate and the false-positive rate. The correct classification of a mammogram is measured by the accuracy. The mathematical expressions of these performance evaluation metrics are as follows:

(4) $$Sensitivity=\frac{TP}{TP+FN}$$

(5) $$FPi=\frac{FP}{N}$$

(6) $$Accuracy=\frac{TP+TN}{FP+FN+TP+TN}$$

where TP: true positive, TN: true negative, FP: false positive, FN: false negative, *N*: total number of images

### 3.11. Experimental Configuration

In this research work, we performed experiments on a Google Colab GPU, 12 GB RAM, and Windows 10 operating system, and all algorithms were implemented in Python 3.6 using Keras and the TensorFlow deep learning library. The computation time was 40 min for training and testing. All the data augmentation steps were implemented in Python using the CV2 image preprocessing library.

## 4. Experimental Results

### 4.1. Results Comparison between the Proposed Method and the DCNN

**Figure 8:**
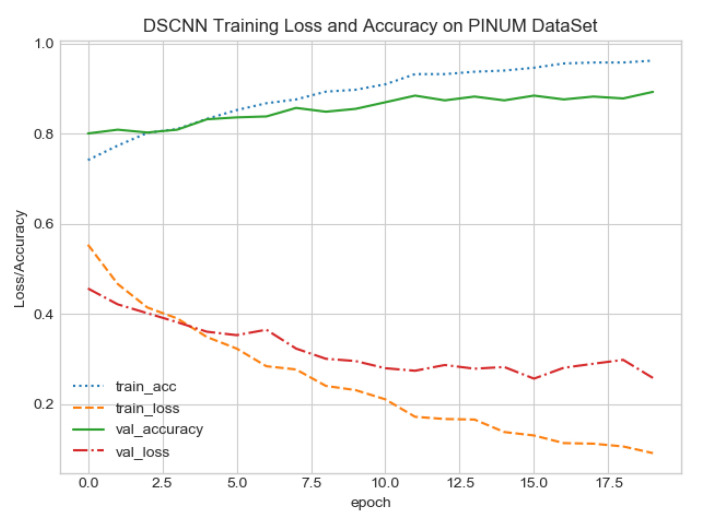
FC-DSCNN training loss and accuracy on the PINUM dataset.

**Figure 9:**
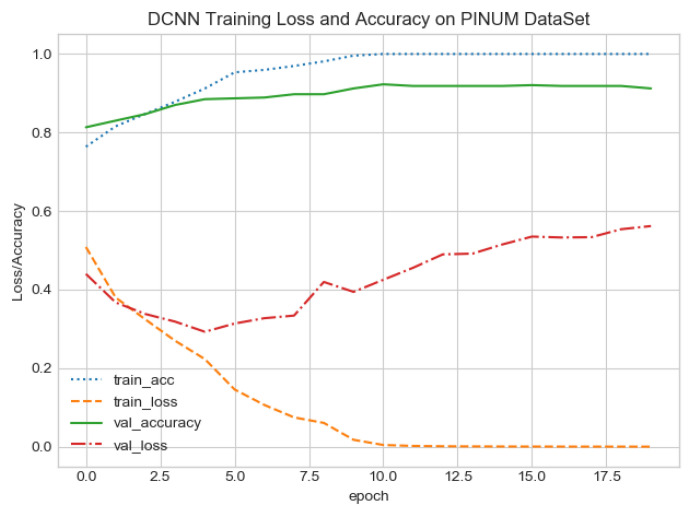
DCNN training loss and accuracy on the PINUM dataset.

**Figure 10:**
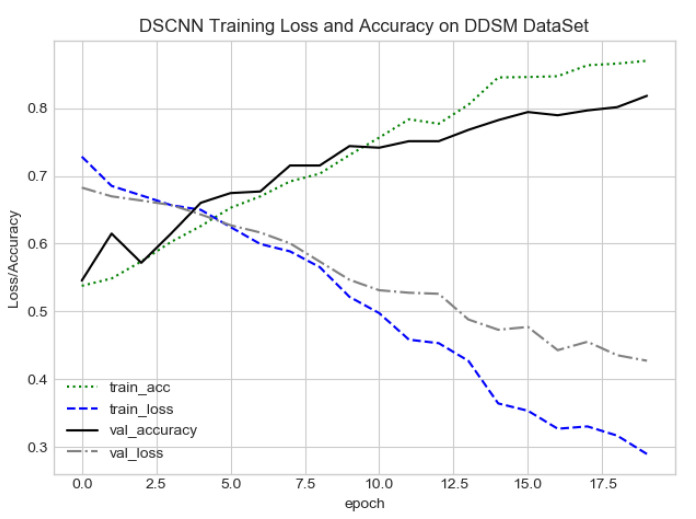
FC-DSCNN training loss and accuracy on the DDSM dataset.

**Figure 11:**
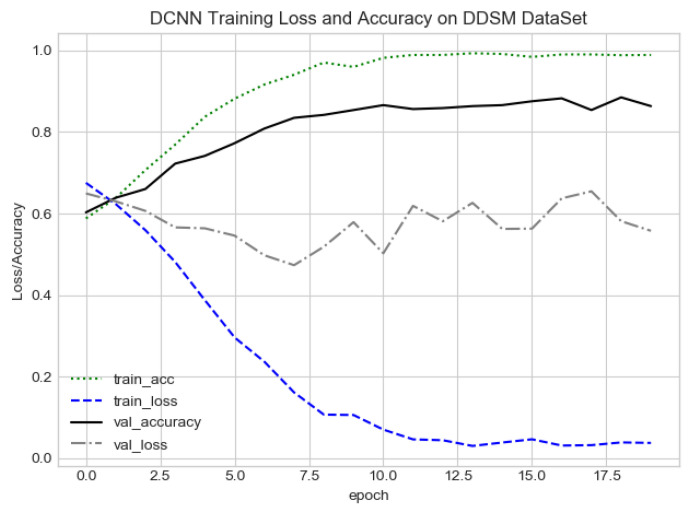
DCNN training loss and accuracy on the DDSM dataset.

The proposed CAD system was designed based on scientific fundamentals for the classification of microcalcification clusters, and we compared the performance of the proposed method with the traditional one. It can be observed that the traditional DCNN shows poor performance compared to the proposed method. We evaluated the classification performance using five-fold cross-validation with the FC-DSCNN and the DCNN for the first time on the local PINUM [51] dataset and on the public DDSM dataset. The performance of the model loss and the accuracy on both datasets is shown in Figure 8, Figure 9, Figure 10 and Figure 11, which represent the loss and accuracy after every 2.5 epochs. In Figure 8, the training accuracy remains higher than the training loss over the iterations, which indicates that our model was perfectly trained. Moreover, we can see that after the seventh epoch, the validation accuracy is consistent, and the validation loss decreases, which indicates that the performance of our proposed network on the PINUM dataset was good and the model was better fitted. In Figure 9, we can see that the training accuracy is still higher than the training loss, while after the seventh epoch, the validation loss increases on the PINUM dataset. The validation loss for our proposed network is lower, and the noise around the main area is 25%, while the validation loss of the traditional neural network increases with 58% noise, which means our network performs much better than the traditional network.

**Figure 12:**
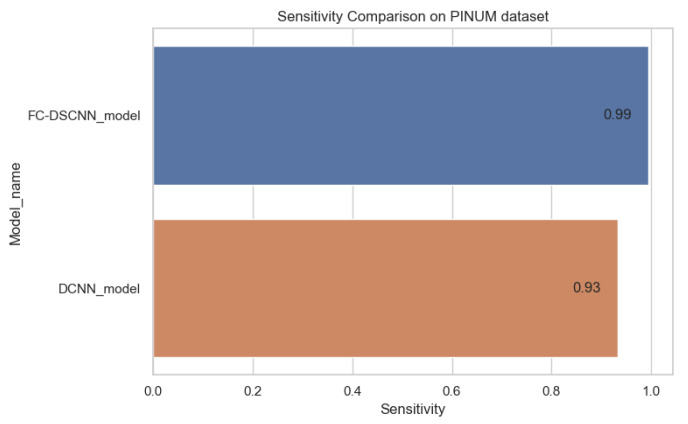
Sensitivity comparison on the PINUM dataset.

**Figure 13:**
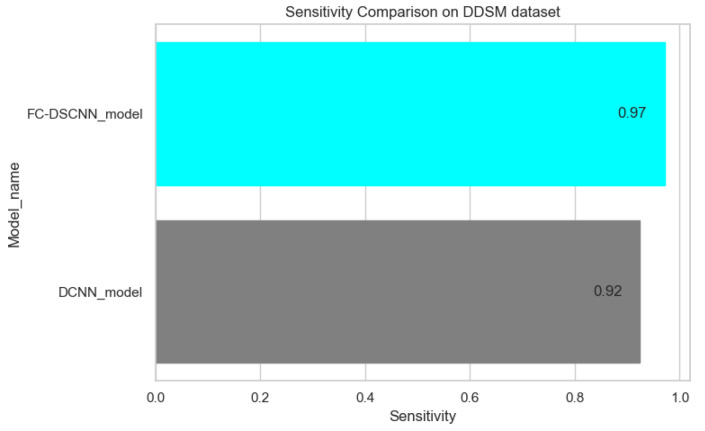
Sensitivity comparison on the DDSM dataset.

The proposed method yields the best performance, achieving a sensitivity of 0.99 with 2.45 FPi and of 0.97 with 2.35 FPi on the PINUM and DDSM datasets, respectively, as shown in Figure 12 and Figure 13. For the DCNN, the maximum sensitivity was 0.93 with a 3.10 FPi and 0.92 with a 2.95 FPi on the PINUM and DDSM datasets, respectively. On the other hand, Figure 10 and Figure 11 reveal that the training accuracy sensitivity of our proposed model and traditional network on the DDSM data is higher while the training loss remains lower over the iterations. The validation loss of our proposed model decreases after the seventh epoch on the DDSM dataset, while the validation loss of the traditional network increases. The performance of our network is much better than the traditional one on the DDSM dataset.

**Figure 14:**
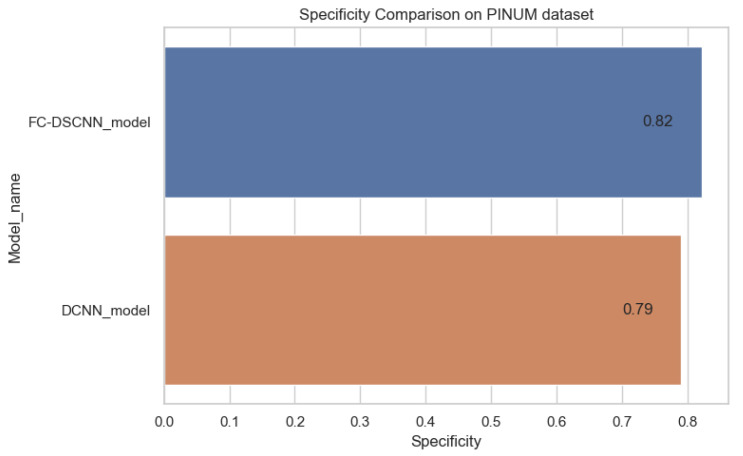
Specificity comparison on the PINUM dataset.

**Figure 15:**
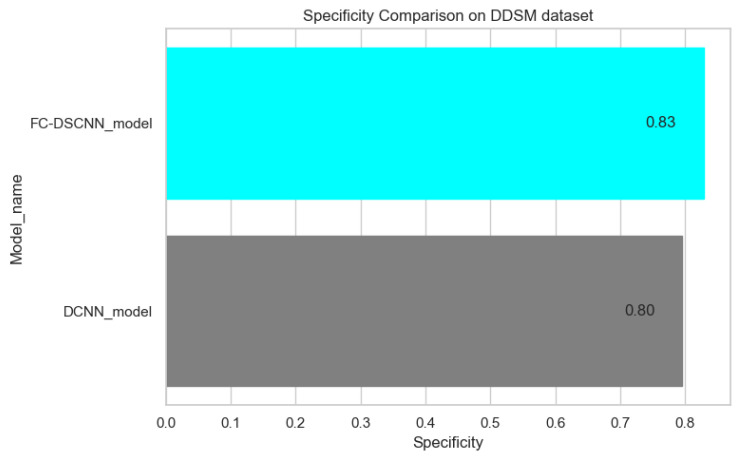
Specificity comparison on the DDSM dataset.

**Figure 16:**
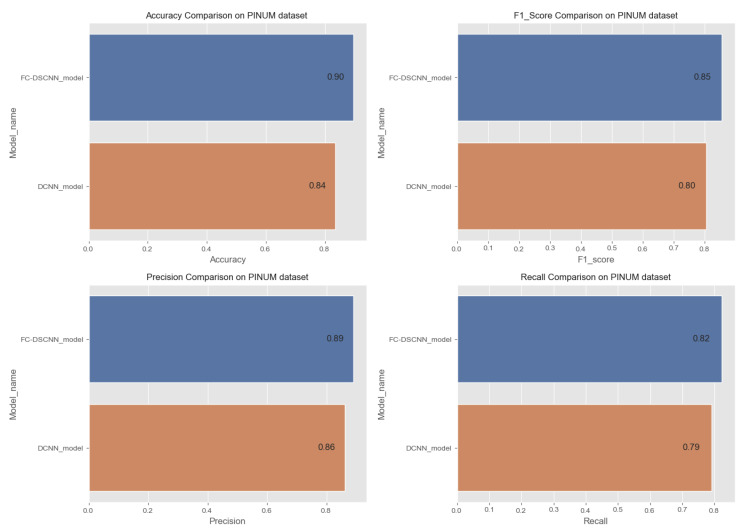
Comparison of the accuracy, F1-score, precision and recall on the PINUM dataset.

**Figure 17:**
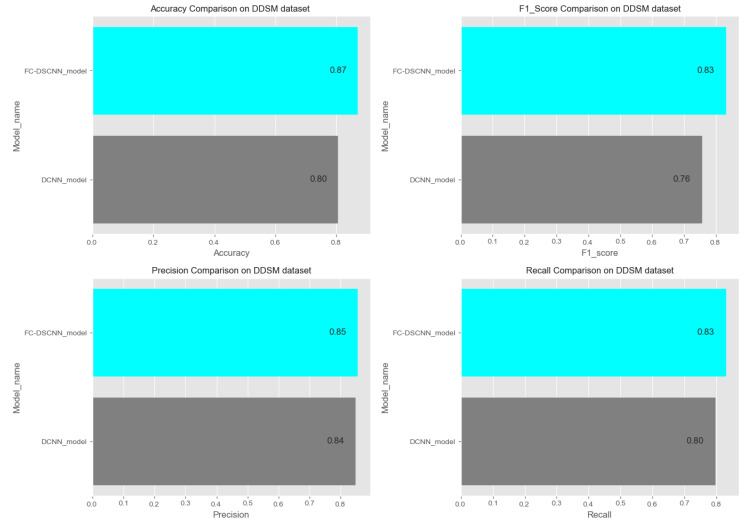
Comparison of the accuracy, F1-score, precision and recall on the DDSM dataset.

In Figure 14 and Figure 15, the results indicate that our proposed method, the FC-DSCNN, reaches 0.82, 0.90, 0.85, 0.89, and 0.82 in the specificity, accuracy, F1_score, precision, and recall, respectively, on the PINUM dataset compared to the DCNN, which has 0.79, 0.84, 0.80, 0.86, and 0.79 in the specificity, accuracy, F1_score, precision, and recall, which is low using identical data. Figure 16 and Figure 17 represent the accuracy, F1_score, precision, and recall on the DDSM dataset, which indicates that the performance of the DCNN compared to the proposed method is very low. The complete summary of the evaluation metrics such as the sensitivity, specificity, accuracy, F1-score, precision, and recall is summarized in Table 4.

Additionally, the comparison of the specificity, accuracy, F1-score precision, and recall for the two methods is listed in Table 4, which as discussed above, enhances the effectiveness of models. It is observed that when the value of the sensitivity is 0.99 on the PINUM dataset with our proposed model, the corresponding values of the specificity, accuracy, and F1-score are higher at 3%, 6%, and 5%, respectively, compared to the DCNN. Moreover, the precision and recall values of the proposed method are 3% and 3% higher than for the DCNN. When the sensitivity increases to 0.97 on the DDSM dataset with our proposed method, the values of the specificity, accuracy, F1-score, precision, and recall increase by 3%, 7%, 7%, 1%, and 3% compared to the DCNN, respectively.

All above aforementioned deep analyses on both datasets reveal that the performance of our proposed method was better. Furthermore, the proposed method demonstrates the usefulness of a deep convolutional neural network in the classification of mammogram images to predict breast cancer at the early stages. Moreover, to validate our results, we evaluated all the performance metrics on the public dataset and compared them on both models.

### 4.2. Results Comparison of the Proposed Method and Previous Studies

To validate our CAD system, we compared it with previous studies by evaluating the local PINUM and the public dataset DDSM. The performance of the proposed CAD system for the detection of microcalcification clusters and classification into benign and malignant classes is best compared to the previous studies. Table 5 reveals that our proposed FC-DSCNN achieves the highest performance with a sensitivity of 0.99 at a 2.45 FPi and 0.97 at a 2.35 FPi on the PINUM and DDSM datasets, respectively.

**Figure 18:**
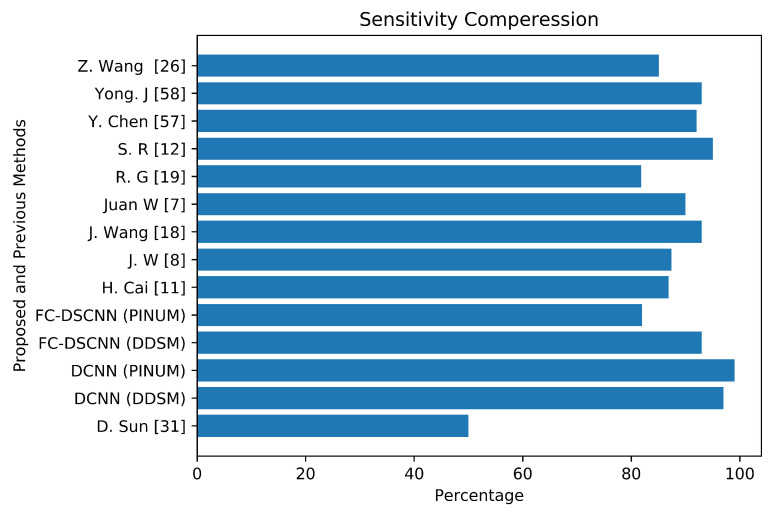
The comparative analysis between the proposed and previous studies showing that the proposed method achieves the maximum sensitivity.

The author’s Juan W. et al. [7], Y. Yang et al. [8], and G. Valvano et al. [10] employed global detection, context-sensitivity, and CNN microcalcification detection deep learning techniques by training on (188,521), (188,521), and 283 mammograms, reporting sensitivities of 90% at a 1.17 FPi, 87.4% at a 1.10 FPi, and a 0.005 FPR, respectively. The deep Learning microcalcification detection approaches by H. Cai et al. [11], S. R. S et al. [12] and J. Wang et al. [18] achieved sensitivities of 86.89%, 95%, and 0.93, respectively, by training on 990, 322, and 1204 mammograms. Another study by R. G. C et al. [19], which did not use deep learning for microcalcification detection, reported a sensitivity of 81.8% at a 2.55 FPi by training on 200 DDSM datasets. The authors Z. Wang et al. [26], D. Sun et al. [31], and R. Shen et al. [40] applied a deep convolutional neural network for the detection of breast cancer by training on 400, 1980, and 1912 images and achieved sensitivities and TPRs of 85.10, 0.50, and 0.96, respectively. Y. Chen et al. [57] employed fine-tuning of the ResNet deep neural network for breast mammogram classification on 2620 DDSM images and achieved 93.83%. Yong J. S. et al. [58] proposed a deep learning model using EfficientNet and DenseNet to classify digital mammograms and obtained 88% and 82% sensitivity, respectively. Our proposed method, the fully connected depthwise-separable convolutional neural network, shows a 0.99 sensitivity at a 2.45 FPi and 0.97 at a 2.35 on 2885 PINUM images and 3568 DDSM images. We observed that our model’s performance is better than the previous studies; the highest sensitivity is 93.83% [57] with a minimum of 81.8% [19] on the DDSM dataset. The highest sensitivity on the private dataset was 0.93 [18], and the lowest was 0.50 [31]. We achieved a 0.99 sensitivity on the private dataset and 0.97 on the DDSM dataset, being about 6% and 4% higher, respectively, than previous studies. Figure 18 presents a sensitivity compression between the proposed and previous studies.

## 5. Discussions

In this study, we propose a state-of-the-art computer-vision-based fully connected depthwise-separable convolutional neural network CAD system for the detection of microcalcification clusters from digital mammograms and classification into benign and malignant classes. The breast cancer classification framework has four steps: image preprocessing and augmentation, microcalcification ROI segmentation, RGB to grayscale channel conversion, and microcalcification cluster classification to predict malignant and benign cases. In our proposed model, we split our dataset into a training set, testing set, and validation set, with 60% of the breast mammogram images selected for training, 20% for testing, and 20% for validation. After the data augmentation, the PINUM dataset contained a total of 2885 digital breast mammogram images at a size of 320 × 240 pixels, both CC and MLO views, from which 2125 were negative and 760 positive images. The DDSM dataset consists of 3568 images including 1740 benign and 1828 malignant images.

**Figure 19:**
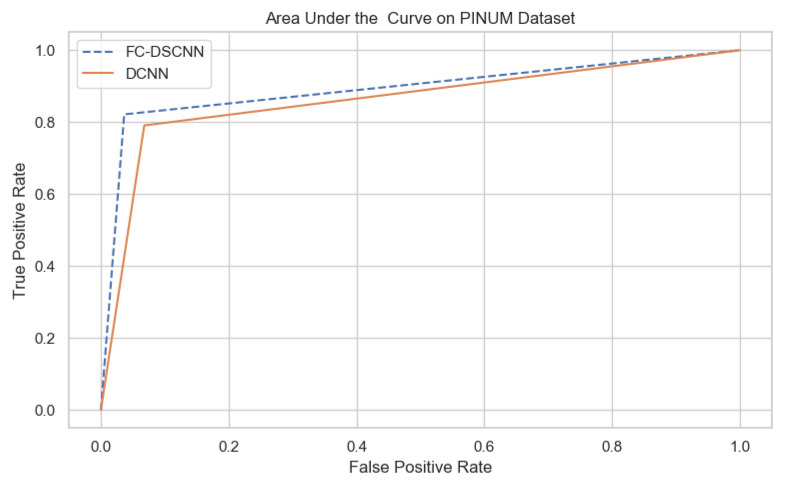
The AUC curve of the proposed method on the PINUM dataset is 0.87, which is higher than the DCNN, which is 0.80.

**Figure 20:**
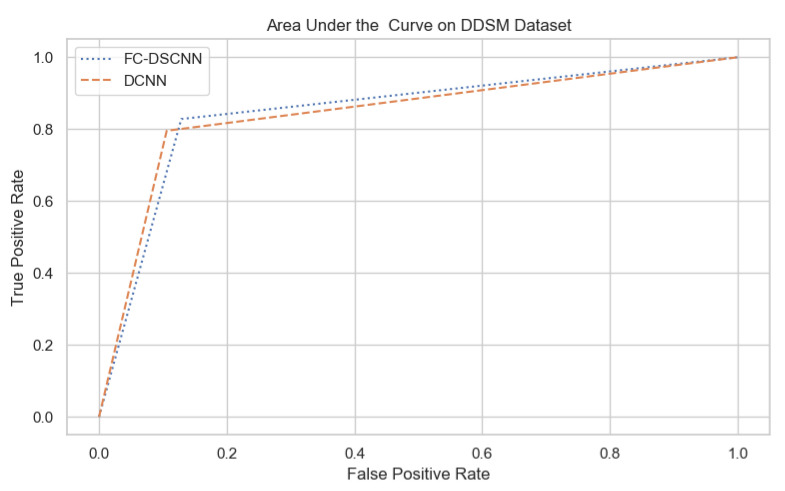
The AUC curve of the proposed method on the DDSM dataset is 0.86, which is higher than the DCNN, which is 0.79.

For our proposed method, the FC-DSCNN, we implemented the best hyperparameter settings: a batch size of 32, a learning rate of 0.001, a dropout of 0.5, and a class weight range of [−1, 1], as presented in Table 6. The training and testing data were split into a 60%, 20% ratio, and the other parameters such as the number of epochs (20), random seeds (42), target size ([320, 240]), sparse_categorical_crossentropy loss function, and the optimization function were degraded. The experimental results revealed that the proposed method significantly outperforms the DCNN and previous studies and achieves a 0.99 sensitivity with a 2.45 FPi and 0.97 at a 2.35 FPi on the PINUM and DDSM datasets, respectively. The other evaluation metrics, such as the specificity, accuracy, F1-score, precision, and recall, are still higher than the traditional DCNN. As seen in Table 4 the sensitivity is 6% greater than the traditional DCNN. The area under the AUC curve of the proposed method and DCNN is 0.87 and 0.80 on the PINUM dataset and 0.86 and 0.79 on the DDSM dataset, respectively, as shown in Figure 19 and Figure 20. Furthermore, the other deep neural networks such as ResNet [57] and EfficientNet [58] with the traditional approaches comparatively show the lowest performance.

Our study shows that the deep learning with the depthwise-separable CNN technique is better at the prediction of microcalcifications compared to the traditional one and the previous studies by Wang J et al. [7], and it outperforms them by achieving the maximum sensitivity. Another deep-learning-based approach reported in [8] achieved an 87.4% sensitivity with a 1.10 false-positive rate. This proposed CAD method was developed for the segmentation of microcalcification clusters from digital mammograms and then classifying them into malignant and benign mammograms. The proposed CAD system is able to predict microcalcifications with accurate results and can encourage radiologists to predict breast cancer at the early stages to save patients’ lives. The handcrafted feature extraction techniques require much time, which decreases the evaluation performance of these models; therefore, the deep-learning-based approaches automatically extract features from images and then perform the classification, which increases the evaluation performance and cancer detection rate.

In this study, we evaluated for the first time the local PINUM [51] dataset and the public DDSM dataset by using deep learning approaches, which increases the rate of the true-positive cases. The evaluation of the local dataset could help doctors and radiologists in the diagnosis of breast cancer in women at the initial stages in real time. Image classification using the deep learning neural network, as a rule of thumb, requires 1000 images per class, and this number can go down significantly if one uses pretrained models [59]. Deep learning neural networks use a data-driven approach, which requires many images to train the network; therefore, to avoid overfitting, we trained with a higher number of images than the previous studies in Table 5. Our proposed model with depthwise-separable convolutions is a novel approach that is applied for the first time to detect microcalcification clusters from mammogram images, achieving a remarkable improvement in the true-positive rate compared to the traditional one and with previous studies. This study employed the computer vision method for locating clustered MCs, as discussed above. However, in the future, we will further investigate the detection of individual MCs using computer vision. Moreover, we will investigate deep neural networks using alternative architectures such as ResNet [57] and EfficientNet [58] with the proposed computer-vision-based model to classify individual MCs on a large dataset. In addition, we will also analyze our model to improve the classification accuracy on 2D and 3D mammograms. The PINUM dataset was evaluated for the first time for research purposes; therefore, the data imbalance issue will be further investigated in future studies.

## 6. Conclusions

Mammography is a standard screening method for the early detection of breast cancer. However, it is not a golden standard for the diagnostics of breast cancer; therefore, it is very difficult for radiologists to provide an accurate prediction of breast cancer at the early stages due to several factors. The early detection of breast cancer and classification with regards to a particular lesion such as microcalcification clusters is a challenging task due to their tiny size. To overcome this issue, we developed a fully automated deep learning model for the segmentation of microcalcification ROIs from digital mammograms and performed the classification for the prediction of breast cancer at the early stages. The proposed method employs a state-of-the-art technique, a depthwise-separable deep convolutional neural network, for the classification of microcalcification clusters into malignant and benign classes. The experimental results revealed that the proposed method outperforms the traditional method and previous studies in terms of increasing the sensitivity and reducing the FPi.

## Figures and Tables

**Table 1:** Dataset description and detail.

Mammogram Grade	Mammogram Label	Resolution	No. of Images	Dataset
0	Benign	4096 × 2047	425	PINUM
1	Malignant	4096 × 2047	152	PINUM
0	Augmented Benign	320 × 240	2125	PINUM
1	Augmented Malignant	320 × 240	760	PINUM
0	Benign	320 × 240	1740	DDSM
1	Malignant	320 × 240	1828	DDSM

**Table 2:** FC-DSCNN structure used for the classification of each layer.

Network Operations	Network Layers	Number of Filters	Filter Size	Stride Value	Output Image
Input image	-	-	-	-	240 × 320 × 3
Convolutional Layer	Separable_Conv (ReLU)	32	3 × 3 × 32	1 × 1	240 × 320 × 32
Batch Normalization	Axis = ChannelDim	-	-	-	240 × 320 × 32
Pooling Layer	Max pooling	1	2 × 2	0	120 × 160 × 32
Convolutional Layer (two times)	Separable_Conv (ReLU)	64	3 × 3 × 64	1 × 1	120 × 160 × 64
Batch Normalization (two times)	Axis = ChannelDim	-	-	-	120 × 160 × 64
Pooling Layer	Max pooling	1	2 × 2	0	60 × 80 × 64
Convolutional Layer (three times)	Separable_Conv (ReLU)	128	3 × 3 × 128	1 × 1	60 × 80 × 128
Batch Normalization (three times)	Axis = ChannelDim	-	-	0	60 × 80 × 128
Pooling Layer	Max pooling	1	2 × 2	0	30 × 40 × 128
Fully Connected	Flatten Layer	-	-	-	153,600
Dropout Layer	Dropout = 0.5	-	-	-	153,600
Fully Connected	Dense Layer	256	-	-	153,600 × 256
Classification Layer	Softmax	-	-	-	2
Classification Out	Classification	-	-	-	2 (Benign/Malignant)

**Table 3:** The DCNN structure used for the classification.

Network Operations	Network Layers	Number of Filters	Filter Size	Stride Value	Output Image
Input image	-	-	-	-	240 × 320 × 3
Convolutional Layer	Conv2D (ReLU)	32	3 × 3 × 32	1 × 1	240 × 320 × 32
Batch Normalization	Axis = ChannelDim	-	-	-	240 × 320 × 32
Pooling Layer	Max pooling	1	2 × 2	0	120 × 160 × 32
Convolutional Layer (two times)	Conv2D (ReLU)	64	3 × 3 × 64	1 × 1	120 × 160 × 64
Batch Normalization (two times)	Axis = ChannelDim	-	-	-	120 × 160 × 64
Pooling Layer	Max pooling	1	2 × 2	0	60 × 80 × 64
Convolutional Layer (three times)	Conv2D (ReLU)	128	3 × 3 × 128	1 × 1	60 × 80 × 128
Batch Normalization (three times)	Axis = ChannelDim	-	-	0	60 × 80 × 128
Pooling Layer	Max pooling	1	2 × 2	0	30 × 40 × 128
Fully Connected	Flatten Layer	-	-	-	153,600
Dropout Layer	Dropout = 0.5	-	-	-	153,600
Fully Connected	Dense Layer	256	-	-	153,600 × 256
Classification Layer	Softmax	-	-	-	2
Classification Out	Classification	-	-	-	2 (Benign/Malignant)

**Table 4:** Performance evaluation of the proposed method and the DCNN.

Method	Sensitivity	Specificity	Accuracy	F1-Score	Precision	Recall	FPi	Dataset
Proposed Method	0.99	0.82	0.90	0.85	0.89	0.82	2.45	PINUM
DCNN	0.93	0.79	0.84	0.80	0.86	0.79	3.10	PINUM
Proposed Method	0.97	0.83	0.87	0.83	0.85	0.83	2.35	DDSM
DCNN	0.92	0.80	0.80	0.76	0.84	0.80	2.95	DDSM

**Table 5:** Comparison of the results with previous studies and the proposed method.

Authors	Methods	Dataset	Images	Sensitivity	FPi
Juan W. et al. [7]	Global detection approach for clustered microcalcification	FFDM, SFM	188, 521	90%	1.17
J. W. and Y. Yang [8]	A context-sensitive deep learning approach for microcalcification detection	FFDM, SFM	188, 521	87.4%	1.10
G. Valvano et al. [10]	Convolutional neural networks for the segmentation of microcalcification	Private data	283	N/A	0.005FPR
H. Cai et al. [11]	Breast microcalcification diagnosis using deep convolutional neural network	Private data	990	86.89%	N/A
S. R. S et al. [12]	Detection and classification of microcalcification from digital mammograms with the firefly algorithm	MIAS	322	95%	N/A
J. Wang et al. [18]	Microcalcification on mammography by deep learning	Private data	1204	0.93	N/A
R. G. C et al. [19]	Independent component analysis to detect clustered microcalcification	DDSM	200	81.8%	2.55
Z. Wang et al. [26]	Breast cancer detection using an extreme learning deep neural network	Private	400	85.10	N/A
D. Sun et al. [31]	Breast cancer diagnosis using a deep neural network	Private	1980	0.50	N/A
R. Shen et al. [40]	Mass detection using a deep active learning neural network	DDSM	1912	N/A	0.96TPR
Y. Chen et al. [57]	Mammogram classification using fine-tuning of ResNet	DDSM	2620	93.83%	N/A
Yong J. S. et al. [58]	Breast cancer detection using EfficientNet and DenseNet	Private	1501	82%	N/A
DCNN	Microcalcification detection with Deep CNN	DDSM	3568	0.92	2.95
DCNN	Microcalcification detection with Deep CNN	PINUM	2885	0.93	3.10
Proposed Method	Microcalcification detection with fully connected depthwise-separable CNN	DDSM	3568	0.97	2.35
Proposed Method	Microcalcification detection with fully connected depthwise-separable CNN	PINUM	2885	0.99	2.45

**Table 6:** Hyperparameter configuration detail.

Configuration	Values
Batch size	32
Learning rate	0.001
Dropout	0.5
Epochs	20
Optimization function	AdaGrad
Class weight	[−1, 1]
Target size	[320, 240]
Random seed	42
Loss function	sparse_categorical_crossentropy
Training split	0.8
Validation split	0.1

## Data Availability

The DDSM [52] dataset is publicly available, and the private PINUM [51] dataset was collected from a local hospital.
